# Editorial: Pharmaceutical Innovation After World War II: From Rational Drug Discovery to Biopharmaceuticals

**DOI:** 10.3389/fphar.2019.00834

**Published:** 2019-07-25

**Authors:** Apostolos Zarros, Tilli Tansey

**Affiliations:** History of Modern Biomedicine Research Group, School of History, Queen Mary University of London, London, United Kingdom

**Keywords:** pharmaceutical innovation, history of pharmacology, drug discovery, biopharmaceuticals, drug repurposing, twentieth century, pharmaceutical industry

The late nineteenth century witnessed unprecedented advances in biomedical sciences, with the promotion in particular of physiological experimentation, bacteriological investigation, and chemical research (Bynum, 1994). These fields expanded and diversified after World War II contemporaneously with technological innovations, increased educational opportunities, the expansion of communication networks, and the creation of a wide range of healthcare markets, from private commercialized arrangements to nationalized organizations. Concomitant with and reflective of these changes were increased expectations of health and wellbeing, and the belief that medical advances could prevent, alleviate, treat, and/or cure physical and mental ills (Hardy and Tansey, 2006).

In the period under review, we have seen, *inter alia*, the shift from traditional *materia medica* to laboratory- and industry-based research; the establishment of the chemical and biological foundations of pharmacology; the gradual transformation from empirical drug discovery to rational drug design; the development of a wide variety of new drugs, as well as—more recently—the impact of molecular biology; and the re-evaluation of therapeutic strategies and priorities (Landau et al., 1999; Sneader, 2005; Church and Tansey, 2007). Thus, as products of and as agents for these advancements, drugs have become fundamental components of modern Western consumer society and of medicine and medical practice in particular.

The aims of this Research Topic were to delineate and conceptualize pharmaceutical innovation in the post-World War II era, and to highlight its roots and pathways throughout that period. Clearly, it has been impossible to include all the relevant themes, topics, and approaches that such an ambition suggests, and we have therefore selected pertinent themes of particular contemporary relevance. Participating authors have contributed to the analysis of the historical and scientific breakthroughs that have shaped pharmaceutical innovation, with an emphasis on the many journeys from rational drug discovery to biopharmaceuticals. These have included the investigation of a variety of paradigm shifting factors, such as the development and acceptance of innovative ideas; the impact of contemporary discoveries, theoretical advances, and technological developments; the influence of individual scientists; the significance of specific pioneering labs; and the varied and variable roles of industry, funding bodies, regulators, and markets.

Godfraind provides a lengthy and informative review of the discovery and development of calcium channel blockers, starting with the observation in the late nineteenth century by Sydney Ringer that calcium was required for cardiac muscle contraction, and then progressively presenting the developments that shaped our understanding of the role of intracellular calcium in muscle contraction. From this work came studies of drugs that blocked calcium entry into cardiovascular tissues—the so-called “calcium channel blockers”—and the recognition of their therapeutic utility in a range of cardiovascular disorders. He concludes his review with a provocative suggestion, based on work on experimental animals, that calcium channel blockers may have long-term effects beyond the cardiovascular system that could be important in human pharmacotherapy.

A similar far-ranging approach is taken by Sanger and Andrews to the discovery of drugs treating nausea and vomiting; their comprehensive review not only discusses the role of serendipity underlying the relevant discoveries but also provides a critical analysis of the role of the pharmaceutical industry in both promoting and hindering drug discovery and implementation. Important issues they address are the repurposing of drugs used for the treatment of nausea and vomiting, particularly during palliative care, as well as the challenges of identifying novel anti-emetic drugs—a field in which there has not been a major breakthrough since the end of the twentieth century.

Quirke’s original research article on tamoxifen as a case study in pharmaceutical innovation follows a different approach by analyzing contemporary archival records from pharmaceutical companies involved in its transition from a failed contraceptive to a best-selling chemotherapeutic for breast cancer. The history of tamoxifen as described in this paper illustrates the limits of the rational drug design, emphasizes the significance of human actors (especially Arthur Walpole of ICI), and sheds more light upon the feedback loops that exist between bench and bedside, which are stereotypical of much pharmaceutical innovation.

A different approach is taken by Al-Humadi et al. in their disease-focused review on the challenges of treating tuberculosis, with a specific emphasis on the decades between World War II and the early twenty-first century. In addition to summarizing the historical sequence of relevant drug development and use, the authors consider issues of drug resistance, drug repurposing, and problems of co-infection as factors influencing the fight against tuberculosis.

Falzone et al. consider pharmacological treatments for cancer at the beginning of the twenty-first century, against the background of a series of turning points defining modern oncology: radiotherapy; the introduction of broad-based chemotherapy; targeted therapeutics; and the recent adoption of checkpoint inhibition as a novel immunomodulatory anticancer approach. Their review emphasizes the constantly changing and evolving field of drug discovery in the fight against cancer, with a focus on the biopharmaceuticals.

A similar discussion on biopharmaceuticals is presented by Li et al. in their review of drugs for autoimmune inflammatory diseases, in which they describe the therapeutic trajectory from small-molecule compounds to anti-tumor necrosis factor (anti-TNF) biologics. In addition to analyzing the historical development of anti-inflammatory drugs after World War II, the authors consider the promise of novel TNF receptor-targeting entities that have already revolutionized the management of autoimmune diseases such as rheumatoid arthritis.

The final contribution by Gu and Pei is a shorter perspective on historical aspects of Chinese herbal medicine (CHM) ranging from traditional practice to scientific drug discovery, with a focus on the antimalarial compound artemisinin. The authors suggest that a new understanding of traditional health practices using modern systematic and *in silico* techniques such as analytical chemistry, systems biology, network pharmacology, and computational modeling could provide promising new interpretations and applications of CHM.

The articles included here cover only a small section of this huge subject of post-World War II pharmaceutical innovation. We hope that these papers will stimulate readers to delve further and reflect on the vast and varied relevant literature.

## Author Contributions

Both authors have contributed equally to this work.

## Conflict of Interest Statement

The authors declare that the research was conducted in the absence of any commercial or financial relationships that could be construed as a potential conflict of interest.
